# Abstracts and Gleanings

**Published:** 1883-01-20

**Authors:** 


### ABSTRACTS AND GLEANINGS.

**Idiocy.**—Dr. Davis, in a paper on the subject of idiocy (Virginia Medical Monthly) says: Idiocy has been classified by many writers, but that of Seguin is the most simple: "The chronic affection of a whole or a part of the central nervous masses, which is characterized as *profound idiocy.* A partial or total affection of the nervous apparatus which ramifies through the tissues and presides over the life of relation, the result of which is *superficial idiocy.*" He also describes a class of cases under the head of "Backward Children," in whom there seems to be a mere functional torpidity of the nervous system.

Dr. Down, in a paper on "The Obstetrical Aspects of Idiocy,"* observes that, in twenty per cent. of the cases of idiocy collected by him, there was a history of marked disturbance of physical health of the mother during pregnancy; in four per cent., a history of serious falls, followed by alarming uterine hemorrhage; in six per cent., prolonged ill health; in ten per cent., persistent sickness, which had occasioned anxiety. He regards vomiting during gestation as an important producer of idiocy. Again, in as many as thirty-two per cent. of the cases, there was, as regards the mother, a history of fright, intense anxiety or great emotional excitement.

Dr. Down † has made inquiries into the causes of idiocy in two thousand cases, and found that in forty-five per cent. there were well marked neuroses in the families of one or both parents.

Dr. Seguin says that not one in a thousand has been entirely refractory to treatment; not one in a hundred who has not been made happy and healthy; more than thirty per cent have been taught to conform to social and moral law, and rendered capable of order, of good feeling, and of working like the third of a man; more than forty per cent. have been capable of the ordinary transactions of life under friendly control, of understanding moral and social abstractions, of working like two-thirds of a man; and twenty-five per cent. come nearer and nearer to the standard of manhood, till some of them will defy the scrutiny of good judges, when compared with ordinary young women and men.

Goltz found, in his recent investigations, that when he removed a great portion of the hemisphere on one side, the animal did not become idiotic or demented; but if he removed portions from either hemisphere, the animal showed a diminution of mental power, with loss of tactile sensibility and awkwardness in its movements. Dr. Ireland,* therefore, supposes that in idiots who improve under instruction, where the whole cerebrum is diseased, it in great part recovers its tone by being brought into healthy exercise, and that where a part still remains sound, it is thrown into more vigorous exercise than the rest, attracts a greater supply of

*Transactions of the Obstetrical Society of London, Vol. XVIII.

†British Medical Journal, Oct. 11, 1873.

*Edinburgh Review, June, 1882.

blood, and gains a more vigorous nutrition than the surrounding parts.

Where the deficiency is congenital, the prognosis is often better than where it is traceable to diseases occurring in childhood; great improvement in the intelligence often follows improvement in the general health, as in the successful treatment of strumous and anæmic conditions.

Without training and education, idiots are an unproductive class; with it, their industrial capacity is greatly increased. The most zealous efforts of earnest teachers in the ordinary schools are ineffectual to meet the requirements of idiots, and it is only by teachers specially skilled and qualified for the duty that these unfortunates can be benefitted, and under their care, in many cases, astonishing moral, intellectual and physical improvement can be accomplished.

**Aspiration of Bladder—Enlarged Prostate—Death.**—A man over 50 years ot age, suffering from retention of urine from enlarged prostate, came under observation. Different forms of prostatic catheters, including the soft rubber, were used, but without effect. When the catheter was introduced it was firmly grasped by the prostatic urethra, and on withdrawal the eyes of the instrument were found filled with blood. These two facts, viz: immobility of the catheter when introduced deeply, together with bleeding on withdrawal, gave rise to strong presumption of a false passage. It was then decided to aspirate in order to relieve the patient of his most urgent symptom.

Potain's instrument was employed, and on withdrawal suction was kept up to prevent any urine from escaping from the needle into the cellular tissue. On the following day the catheter was again employed, but without success, and aspiration was again performed. Symptoms of peritonitis set in shortly afterwards, and on the following day the patient died. The autopsy showed a very much enlarged prostate, but no evidences of a false passage were noticed. There was no sign of peritonitis around the needle punctures, and very slight evidences of inflammation in any part of the peritoneal cavity. The bladder contained only a few ounces. There was no extravasation of urine. The case was interesting and painfully unpleasant and unsatisfactory in its termination. The autopsy unfortunately did not throw much light on it.

Shortly after the case was seen, another one, of enlarged prostate, with retention, came under observation. Several attempts were made to pass the catheter, but, as in the case recorded, the instrument was firmly held by the prostate, and on withdrawal the eyes were filled with blood. A final attempt was made and considerable force used, when the catheter passed the obstruction, and the anxious patient was gratified by a copious flow of urine.

These two cases are of more than common interest in regard to the treatment of retention of urine in cases of enlarged prostate, and the inference is that it is better to use a certain amount of force, at the risk of making a false passage, than to expose the patient to the dangers coincident to a distended bladder.

### PUZZLING CASE OF INCARCERATED HERNIA—OPERATION—DEATH.

A man, 35 years of age, was seen after he had been suffering for a few days from what was supposed to be strangulated hernia. The patient gave the usual history of constipation and abdominal pain. An examination showed a peculiar swelling in the inguinal canal, not unlike what might be expected from hydrocele of the spermatic cord. It was not hard, and the finger could pass up the inguinal canal. It was considered best, on account of the urgency of the patient's condition, to operate and explore the canal. In doing so there was found to be a partially strangulated or incarcerated hernia at the left internal abdominal ring. The puzzling condition of the canal was due to fluid, the result of the hernia. The patient died shortly after the operation.

### SOME OF THE DIFFICULTIES IN ASPIRATION OF THE CHEST.

A man, aged 70 years, suffered from chronic bronchitis, and subsequently from pleurisy and emphysema on the right side. Surgical interference was not attempted till the dyspnœa became oppressive. He was then aspirated, and about two quarts of foul smelling pus removed. Great relief followed the operation, and the patient was allowed to rest for a few days, when he was again aspirated to relieve the distress of breathing.

On the afternoon of the same day it was decided to make a permanent opening and wash out the pleura. The usual incision was made at the point of aspiration, but no pus obtained. Another incision was made lower down and the precaution made to carry it pretty far into the chest, but still not a drop of pus was found. On the following day a swelling appeared below the chest wall, on the same side, but as the patient was moribund, no exploration was attempted. Unfortunately, no autopsy was held.

There are some pertinent questions which readily suggest themselves, such as the whereabouts of the fluid, inasmuch as only a limited amount was removed on the morning of the operation. The facts, unsatisfactory though they be. are respectfully submitted to the readers of the Reporter, with the hope of bringing forward some suggestion.

One thing that painfully impressed itself on my mind was, do not aspirate shortly before making a permanent opening in the chest wall.—*Med. and Surg. Rep.*

**Ergot in Cancer.**—Dr. Collins, in Lancet and Clinic, remarks of ergot as a remedy for epithelial cancer and kindred tumors:

I will not attempt to give the therapeutic reasoning by which I was induced to use the ergot in the first place, but I will say in the first place that I found that all writers on this subject held that these ulcers progressed by reason of their excessive cell growth or hypernutrition and knowing almost if not every remedy had failed of the cure I could think of nothing—as an effort—more suited to the indications, that of diminishing vitality, vascularity and capillary circulation, and at the same time as free from objection, than ergot.

I tried it in the first case and the manifest result was astounding to the patient, other physicians who had treated him and myself, and was as incredibly unexpected as acceptable. To the incredulous I will say that the exact correctness of this report is attested by every medical man who saw the case.

Case I. W. W., æt. 67. Applied to me in April, 1876, for the treatment of an ulcer of the left side of the neck and face, involving the parotid gland and ear; which ulcer was about four or five inches in diameter, extending from the meatus of the ear downward and forward along the sterno cleido mastoid muscle, the center being below the angle of the lower jaw, and was of an irregular circular shape and surrounded by an elevated, indurated and nodulated margin or roll about one inch in diameter, thus making the extreme extent of the ulcer about six inches. The general depth being about one inch below surface of margin, yet in the center it was deeper, at which point the pulsation of the carotid artery was plainly visible. The floor was covered with indurated, nodurated bodies and the discharge was of a sanious nature, the elevated margin and the floor being quite hard. The cervical, post-cervical and sub-maxillary lymphatic ganglia were much involved, being indurated and tender. He was emaciated, weak, had poor appetite, and colliquative sweats, and the color of his face, which in health had been very florid, was very pale and the exact appearance of the cancerous cachexia in advanced cases. The ulcer commenced in a hardened elevated tumor of the skin just below the point of the ear, growing slowly, not painful at first; when, about two years previous, it had attained the size of a wulnut, the ulceration of the surface of the tumor began and pain also set in. The ulceration of the ulcer steadily progressed until it attained the si?e described, and latterly had frequent profuse hemorrhages, until at the time I saw him death seemed imminent from exhaustion of the carotid vessels. The situation and depth of the ulcer precluded the use of the knife or cautery, and all of the physicians whom he had consulted having told him that his death would shortly occur, at his urgent request I undertook to try something and my investigation led me to try ergot for the easons stated.

The fresh orgot was freshly ground to an impalpable powder and applied three times daily to the entire face of the ulcer with a large soft hair-pencil, the ulcer being washed thoroughly once every day. The powder was used dry, allowing all to adhere that would. After each application the ulcer was covered with a light muslin rag wet with a lotion of

| | | |
|---|---|---|
| ℞ Carbolic acid | 1 | drachm |
| Sulphurous acid | 4 | drachms |
| Glycerine | 1 | ounce |
| Aqua | 2½ | ounces |

M. Sig. Lotion.

He was also put upon quinine, iron, cod-liver oil and the other usual adjuncts to a restorative treatment. In a short time the discharge assumed the appearance of laudable pus, the induration of the lymphatic ganglia disappeared, the elevated, stony and nonulated periphery subsided, the floor assumed a healthy aspect, and in twelve weeks the ulcer had entirely healed and has remained so up to the present time. His general health rapidly improved, and up to this time has been continuously good. This gentleman had an uncle to die of a similar ulcer and an aunt of cancer of the breast. All of the physicians who treated this case previous to myself were and are confirmed that it was epithelial cancer.

CASE II. Mrs. E. K., æt. 40, married, no children. This case was sent me from an adjoining county in March, 1877; by her family physician, with the statement that she was suffering from cancer of the left breast of about three years standing. I found the body of the gland entirely destroyed and its site occupied by an excavated ulcer about five inches in diameter and an average depth of about one inch. Margin elevated, indurated and nodulated. The ulceration was in active progression, her general system much depressed, loss of appetite, colliquative sweats, etc. The lymphatic ganglia of the left axilla were enlarged, tender and indurated, in fact were so coalescent with the margin of the ulcer that I could discern no line of separation. The super clavicular glands of the left side were extensive, indurated and enlarged. This case began as the preceding—by a small scaly movable tumor of the skin near the nipple. Her family history showed no case of cancer. The treatment of this case was precisely the same as case No. 1, yet the recovery was not so rapid, and it was not until August following that the ulcer was covered by a cicatrix, and not until December that all induration and tenderness had disappeared, and up to this date it has shown no sign of recurrence. Her general health has also remained good.

CASE III. Andrew B., aged 70. Applied to me in October, 1877, with an ulcer of the outer angle of the left orbit, about two inches in diameter, with an elevated, indurated and nodulated margin. The ulcer being sunken, the floor being covered with tuberculated elevations, the discharge slight and sanious, and the ulcer painful. It was of about twenty months standing, and commenced with a scaly tubercle of the skin, which ulcerated about a year before I saw it. This case had been treated by two excellent physicians before I saw it, both of whom pronounced it epithelial carcinoma. This case was treated by the application of the dry powdered ergot three times daily, washing it once daily and at all times keeping it free from any crust or scab. This ulcer healed nicely in two months, and has shown no sign of return.

CASE IV. Thomas H., æt. 55. Applied to me in March, 1879, with what his physicians had told him was cancer of the lip of one year standing. I found him suffering from a fungoid ulcer of the mucous margin of right side of lower lip, very painful and bleeding frequently. I removed the fungous mass by scissors and applied the ergot as in the preceding cases, which soon produced an excavated ulcer about three-fourths of an inch in diameter, and under the continued use of the ergot the ulcer filled up by granuations, leaving but a slight scar. This patient had an older brother to die from an ulcer of the lip exactly similar to his own as far as it proceeded.

CASE V. M. D. B., æt. 75. Came under my treatment in 1875 with an ulcer of the nose of ten years standing. He had been treated by a number of physicians, but with little benefit. In 1877 I commenced applying the ergot and succeeded in arresting the ulcer in the locality ulcerated, yet it would ulcerate in new places until 1881, when it died.

CASE VI. J. G. C., æt. 45. Applied to me in March, 1880, with an excavated ulcer, one inch in diameter, of the right upper eyelid, of eighteen months standing, which had been treated by three reputable physicians, all of whom pronounced it cancer. His mother died of scirrhus of the breast. The application of ergot was used, and in six weeks the ulcer was healed, and there has been no return.

CASE VII. John S., æt. 65. Applied July, 1880, with carcinomatous ulcer of right side of cheek, near the nose, of two years duration. The case was very similar to case VII, the ulcer being somewhat larger. The ergot was used and perfect healing resulted in two months and no return to date.

CASE VIII. George D., æt. 48. Applied January, 1882. Ulcer of left side of the nose, one inch in diameter, twenty months duration. Healing resulted in five weeks under same treatment.

CASE IX. Joseph H. L., æt. 70. Applied March, 1882. Ulcer of tip of the nose, of three years duration, one and one-half inches in diameter. Treatment as before and recovery in four weeks and no return.

CASE X. John S. W., æt 60. Applied September, 1880. Ulcer of left cheek, two inches in diameter, excavated with raised edges, of two years duration, and very painful. Treatment as before and recevery in four weeks, and no return.

**Subcutaneous Injection in Puerperal Convulsions.**—Dr. R. M. Bowstead, in the British Medical Journal, reports as follows:

Some years ago, I published two cases of puerperal convulsions cured by the subcutaneous injection of morphia with tincture of aconite and solution of atropia; and in 1878, I cured another case by the same means. I think, however, the present case will prove the efficacy of the remedy still more strongly.

I was sent for to attend L. P., aged 29, a primipara, at 1 a. m., on June 12th, 1882. Her legs were œdematous; her urine contained a trace of albumen. There was œdema also of the labia. At 11:15, a. m., she was delivered of a male child. I left soon afterwards, as everything appeared to be satisfactory. At 3 p. m., her husband called to say that his wife was suffering from severe headache and sickness. I sent a saline bismuth draught, which at once relieved her. At 4 p. m., I was hastily summoned, as she was reported to have a fit. On my arrival I found her in strong convulsions, frothing at the mouth. The fits continuing one after another, I injected into her arm half a grain of morphia, two minims of Fleming's tincture of aconite, and two minims of liquor of atropiæ. This had the effect of slightly diminishing the fits; but they continued. After half an hour, I repeated the injection. The fits immediately ceased, and she fell into a sound sleep. At 11 p. m., I saw her again; she was sleeping, and perspiring freely. As she had not passed urine, I introduced a catheter and drew off a pint. The lochia had ceased.

On the 13th, at 9 a. m., she had slept all the night, and was still inclined to sleep, but quite sensible. At 3 p. m., she was sensible, and asked for something to eat. Pulse 120; temperature 104°. She had passed urine freely, but it was not offensive to the smell. The lochia had appeared slightly. At 10 p. m., pulse 110; temperature 101°. She had taken freely of support, but was rather restless. I gave her a draught of chloral hydrate with bromide of potassium.

On the 14th she had passed a tolerable night. The lochia were now profuse. I gave her a minim of tincture of aconite every hour, which caused her to perspire freely. Her bowels were relieved by castor oil. After this she continued to improve; and on the 16th, I gave her a mixture of quinine.

On July 3d, she was convalescent, and able to sit up. The urine was natural. She is naturally delicate, having lost two sisters in childbirth; and the whole family are consumptive.

I publish this case with the hope that other medical men will try this treatment, and be able to write as favorably as I have done. In all my cases the injection has acted like magic. I made the injection in 1868; and its effects are as good as ever.—*New Orleans Med. Journal.*

**Clinical Remarks on Boracic Acid.**—For a long series of years we have been drawing the attention of the profession to what we have been pleased to term a rational treatment of external diseases of the eye, and each year has been marked by decadence of the old astringent-caustic plan.

We believe it was Lister who popularized boracic acid, for a long time a secret remedy in Belgium.

Worlomont claims more benefit from its use in external ocular affections than from all other remedies. It has found other strong advocates, among whom we may mention Panas, of Paris; Theobold, of Baltimore, and Masse, of Bordeaux.

Probably now a large number of oculists use boracic acid more or less, and it is without doubt a remedy of very general application and of great utility. The basis of its action is without doubt its toxic effect on parasitic growths, and hence its value in catarrhal and purulent affections, most clearly demonstrated in purulent otitis media, where the most brilliant results have been shown.

We are not aware that great stress has been laid on its use in blepharitis marginalis, though we know it has been used for this disease. The fact is, since the relation between strain on the eye and blepharitis marginalis has become known, and that the great remedy in connection of the anomaly in structure or balance of muscles, unguents have lost laigely of their importance. All the old pencillings with nitrate of silver, smearing with nasty mercurial salves have practically gone by.

For two or three years in both private and dispensary practice, when we have written for anything in this, it has been three per cent or four per cent of boracic acid in vaseline (15 to 20 grs. to the oz.), and we can vouch for its answering every purpose.

We never treat a case of purulency now without confining the cleansing of the eyes to solutions of boracic acid, and for the milder forms in which we are unable to personally carry out the treatment we allow nothing else. In the severe form we apply once a day the yellow oxide and vaseline as used for so many years by myself, and perhaps in addition, a solution of eserine.

It is quite generally known now that antiseptic precautions have obliterated in both hospital and private practice, the ophthalmia of new-born children, and as a prophylactic we must earnestly advise the exclusive use of boracic acid solutions, and the entire discarding of carbolic acid, since it is extremely annoying and practically demonstrated to be valueless as an antiseptic, and we need not say astringents should be treated in the same way.

Boracic acid, moreover, can claim the virtue of being inert and painless.

It is certainly firmly established now that a true purulent conjunctivitis is due to the presence of a micro-organism definitely fixed and known, and any treatment or remedies to be rational must be parasiticidal. We still adhere (and all investigations confirm the view) to our so often asserted opinion, that mercury is the great antiseptic, but there are conditions and circumstances that render other remedies better applicable. We firmly believe that the strikingly efficient action of mercury in catarrhal conjunctivitis and trachoma, demonstrate the parasitic nature of these affections, though now we have had found for us the microbe of trachoma by Hubert Sattler, of Erlangen.—*Dr. W. W. Seely, in Lancet and Clinic.*

**The Latest About Bacteria.**—This time it comes from America.

Dr. Formad, of Philadelphia, has made some experiments, from which he is led to believe that, contrary to the generally accepted view, bacteria are not the cause *per se* of disease, but are merely the vehicle of contagion, the means by which the poison of certain diseases is carried from one organism to another. He has found the tubercle bacilli of Koch, or, at least, bodies identical with them, in the sputa of non-phthisical patients.

He has taken matter infested with the diphtheritic micrococci (first demonstrated by Professor Wood and himself), and has succeeded in producing the disease in animals, while micrococci from the very same specimen, *after a thorough washing in plain water*, were perfectly harmless.

He believes that bacteria exist in all nature, and that, even when charged with the elements of disease, they cannot produce the disease unless they find a resting place in some body that is, on account of some unexplained conditions, a suitable pasture for the growth and development of the particular disease.

This view, which seems to be a very rational one, has two important practical bearings.

In the first place, it teaches us very forcibly the value of water as a disinfectant, and in the second place, it lends much additional force to the idea that infectious diseases are due to chemical influences, which theory, if demonstrated, will do much toward increasing the potency of our therapeutic resources.

If these bacteria carry certain elements that are capable of producing disease, it is not unreasonable to hope that chemistry will step in and tell us the chemical nature of these poisons; from this standpoint it will be but a step to name the chemical that will prove the antidote, and by this process can we arrive at the most rational treatment of infectious diseases. Dr. Formad promises to have more to say on this subject, and we look forward to his researches with interest, for it would seem that he is on the right track.—*Med. and Surg. Reporter.*

**Angina Pectoris—Death.**—Dr. J. M. Stevenson reports a somewhat interesting case of angina pectoris in the Pittsburg Medical Journal. A man, aged 64, temperate and active, after being engaged in active work all morning, ate a full dinner. Immediately afterwards, with a cry of pain, he sank to the floor, struggling and insensible. Brandy and strong aromatic spirits of ammonia failed to elicit any evidence of their irritant properties. After using a variety of remedies, six drops of nitrite of amyl were poured upon a handkerchief and administered very cautiously, allowing an abundance of air. After a few inhalations the face became flushed, pulse increased in frequency and fullness, respiration became quicker, in ten minutes all struggling ceased, and in twenty minutes consciousness returned. Three days subsequently he had a similar attack which was relieved by the same means, but on the evening of this day he had another and, no one being present to administer the amyl, he died in five minutes. A post mortem was refused.—*Med. and Surg. Reporter.*

**Naphthaline as an Antiseptic.**—Naphthaline has recently found a new and important use in medicine. It has been found that this hydrocarbon is an excellent antiseptic, which kills fungi and bacteria in a short time. For surgical bandages and in contagious diseases, as far as experiments have been made, it has answered an excellent purpose, and seems well adapted to replace, in many cases, those antiseptics now so much used, namely, carbolic and salicylic acids, and iodoform. It has one great advantage over carbolic acid, being absolutely free from poison, and can therefore be used in any desired quantity without causing any disturbance. It also surpasses all other antiseptics in cheapness. As 100 kilos of pure naphthaline can be bought for 60 marks (about seven cents per pound), there is no doubt that it will soon find general use for medical purposes.—*Scientific American.*—*Cincinnati Lancet and Clinic.*

**Bacteria of Syphilis.**—M. M. Martineau and Hamonic (L'Union Medicale) have found the bacteria of syphilis, and have succeeded in inoculating a pig with syphilis from the culture liquid. The bacteria are thus described: they are rod-shaped, of variable length, but not surpassing in length the diameter of a blood globule, formed of a clear matter, and contain no trace of a nucleus, envelopé nor granulations. They are grouped by twos, or are single, or are joined end to end and two by two, but between the conjoined bacteria there is a small, clear space, so that, properly speaking, they are not in contact. Some are joined so as to form, more or less, an open angle, and sometimes three by three. They offer divers movements around a central axis like a compass needle: some pirouette around a transverse axis; others around one of their extremities, which appears fixed; others have an undulatory or serpent-like movement. Numerous other bacteria of varying sizes, forms and movements were seen.

These bacteria, above described, were obtained by immersing an excised, indurated chancre in a flask containing Pasteur's culture fluid. The liquor lost its transparency in three hours: in six a small grey deposit had formed, and in twenty-four hours the bacteria were found and inoculated into a young pig, in whose blood the next day were found analogous bacteria. A control experiment was made by inoculating a second pig with serum from an infecting chancre, and four days after bacteria analogous to those of the first experiment were found in the blood, and shortly afterwards papular syphilides appeared, persisted for many days and finally disappeared two months after the experiment.—*Canadian Journal of Medical Science.*

**New Anæsthetics.**—Dr. V. Mering, at the recent meeting of German Naturalists and Physicians, reported his experiments with two new anæsthetics; diethylacetat and dimethylacetat. The former has a burning, pungent taste, the latter a disagreeable smell and taste. Both produce narcosis very rapidly in frogs and rabbits. There is slowing of the heartbeat, and finally weakening of respiration. In inhalation they act much like chloroform. Mering gave the diethylacetat to some criminals and found that it acted very well, producing narcosis with no bad after-effects.—*Ex.*

**Abortive Treatment of Facial Erysipelas.**—Norregard (Nordisk med. Arch., vol. xii., No. 27) has several times checked the progress of facial erysipelas by drawing a thick ring of collodion around the affected part, not over the whole surface, as by others. Dr. Christie mentions a similar case, in which the swollen skin bulged over the ring without being able to pass the barrier. The ring must be strong, especially on the bearded portion of the face.—*Arch. of Dermatol, October, 1882.*

**According** to calculations made by the Academy of Paris, there are at present 189,000 doctors scattered over the world. Of these 65,000 are in the United States, 26,000 in France, 32.000 in Germany and Austria, 35,000 in Great Britain and its colonies, 11,000 in Italy and 5,000 in Spain.—*Virginia Med. Monthly.*

**Albuminuria in Phthisis.**—Dr. Cailleret, in a well-written thesis (Paris Medical) upon this subject, arrives at the following conclusions:

1. Albuminuria in the tuberculous is found in about one-sixteenth of the cases, according to the different statistics given.

2. Albuminuria is always a serious complication of phthisis; it may be transient or permanent.

3. Transient albuminuria in phthisis, as in all consumptive diseases, is a dyscrasic albuminuria, connected with a renal congestion; when permanent, it is dependent either on a tuberculous, epithelial or interstitial nephritis, or upon an amyloid degeneration of the kidneys.

4. Albuminuria does not seem to influence the progress of pulmonary phthisis, or to hasten the evolution of tubercles.

5. The prognosis is always grave when there is a permanent albuminuria, for there can be no hope of cicatrization of the pulmonary lesions.

6. Milk diet is the one which has given the best results, and which ought to be applied, together with astringents.—*St. Louis Med. and Surg. Jour., November, 1882.*

**Small Doses.**—In concluding this letter I will give you a few of what Prof. Smith, of Bellevue, on Materia Medica, calls his small doses. He distinctly wishes it understood, however, that he is no homeopathist. I do not recollect to have seen them published elsewhere:

Castor oil, five drops, rubbed up with sugar and given every two hours in intestinal irritation of children.

Tinct. hamamelis, one drop every fifteen minutes as a sedative in children.

Tinct. pulsatilla, one drop in desmenrorrhea every fifteen minutes, also in orchitis and epididymitis.

Fowler's solution, one-half drop in nausea of pregnancy and after a drunken debauch.

Tartar emetic, one grain in a quart of water. Dose, one teaspoonful every fifteen minutes in the bronchitis of children.

Calomel, one-fiftieth of a grain in syphilitic headache, without gummata, every fifteen minutes. Also in children with vomiting, accompanied with mucous discharges, one-half grain bichloride of mercury in a pint of water, and administered in teaspoonful doses every fifteen minutes; good for the same affections.

Fl. ext. ergot, one drop every fifteen minutes in menorrhagia.—*Extract of Letter to Medical News.*

**The Development of Vaccinia from Variola.**—The experiments of Dr. Voigt, Superintendent of the Vaccine Institute at Hamburg (Deutsche Vierteljahrsschrift fur Œffentliche Gesundheitspflege. Bd. xiv, Heft 3), have, it is claimed, finally demonstrated conclusively the possibility of transmuting the most virulent variolous pus into vaccine lymph, which possesses all the usual characters, and which is now being used successfully for public vaccination in this Institute.—*Clin. Rec.*

